# Multicompartmental analysis of the murine pulmonary immune response by spectral flow cytometry

**DOI:** 10.1152/ajplung.00317.2022

**Published:** 2023-08-15

**Authors:** Mary Y. Chang, Jourdan E. Brune, Michele Black, William A. Altemeier, Charles W. Frevert

**Affiliations:** ^1^Department of Comparative Medicine, University of Washington, Seattle, Washington, United States; ^2^Center for Lung Biology, University of Washington at South Lake Union, Seattle, Washington, United States; ^3^Department of Immunology, University of Washington, Seattle, Washington, United States; ^4^Division of Pulmonary, Critical Care and Sleep Medicine, Department of Medicine, University of Washington, Seattle, Washington, United States

**Keywords:** compartmental analysis, full-spectrum flow cytometry, heterogeneous autofluorescence, influenza, pulmonary immune response

## Abstract

Studies of pulmonary inflammation require unique considerations due to the complex structure and composition of the lungs. The lungs have multiple compartments and diverse immune cell populations, with inherently high autofluorescence, and are involved in the host response to pulmonary pathogens. We describe a protocol that accounts for these factors through a novel combination of methodologies—in vivo compartmental analysis and spectral flow cytometry with a broad panel of antibodies. In vivo compartmental analysis enables the precise localization of immune cells within the marginated vasculature, lung interstitium, nonlavageable airways, and lavageable airways of the lungs, as well as the pulmonary lymph nodes. Spectral flow cytometry with a broad panel of antibodies supports an unbiased exploratory approach to investigating diverse immune cell populations during pulmonary inflammation. Most importantly, spectral flow uses cellular autofluorescence to aid in the resolution and identification of immune cell populations. This methodology enables the acquisition of high-quality data compatible with informed gating and dimensionality reduction algorithms. In addition, our protocol emphasizes considerations for compartmentalization of the inflammatory response, spectral flow panel design, and autofluorescence spectra analysis. These methodologies are critical for increasing the rigor of pulmonary research. We apply this protocol for the precise characterization and localization of leukocytes in the pulmonary host response to influenza A virus in C57BL/6J mice. In particular, we demonstrate that this protocol improves the quantification and localization of alveolar macrophages within the airways. The methodology is modifiable and expandable to allow for further characterization of leukocyte populations of special interest.

**NEW & NOTEWORTHY** We describe a novel combination of methodologies that incorporates dual in vivo compartmental analysis using intravascular and intratracheal CD45 labeling, a broad panel of antibodies for identifying lymphoid and nonlymphoid cells, and spectral flow cytometry that uses cellular autofluorescence to aid in resolving and identifying immune cell populations. This methodology allows precise localization of immune cells in the lavageable airways, nonlavageable airways, interstitial lung tissue, and marginated in the lung vasculature.

## Graphical abstract

**Figure:**
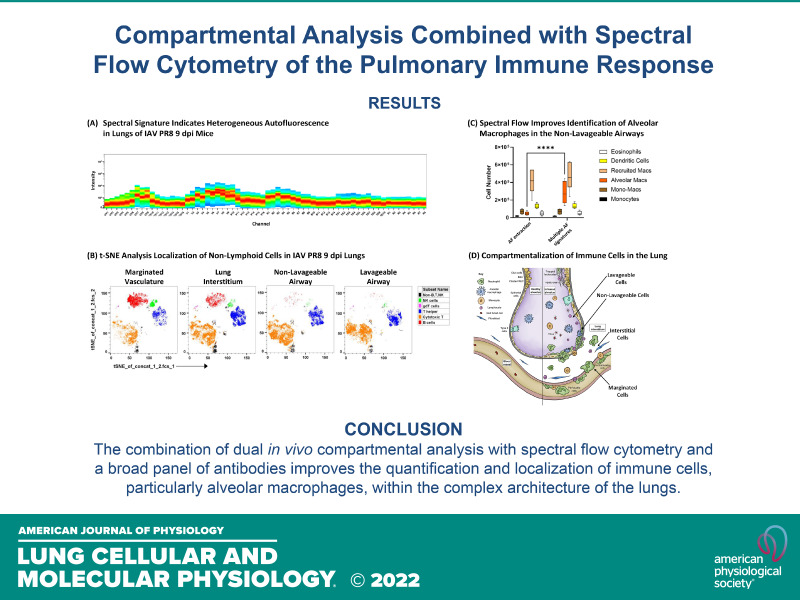

## INTRODUCTION

Leukocyte trafficking in response to infection and injury is central to the pulmonary immune response. When successful, leukocyte recruitment leads to the containment of the threat, followed by clearance of immune cells and resolution of inflammation. However, many lung diseases are characterized by an immune response and leukocyte recruitment that results in severe lung injury or impaired resolution of inflammation. Understanding the fundamental processes underlying the pulmonary immune response has unique challenges due to the complex structure and composition of the lungs, with multiple compartments and leukocyte populations involved in the immune response, depending on the inflammatory stimulus or the kinetics of the host response. This complexity requires special attention to acquire high-quality data that accurately reflect lung pathophysiology. Here, we describe the methodology necessary for accurately measuring the lung’s immune response to the influenza virus by incorporating spectral flow cytometry and in vivo pulmonary compartmental analysis. The protocols described in this manuscript to measure leukocyte migration into the lungs of mice infected with the influenza virus can be easily adapted to other animal models.

Two aspects of spectral flow are well-suited for this approach. First, spectral cytometers collect data from detector arrays covering the entire emission wavelength spectrum. Full-spectrum analysis enables the ability of spectral cytometers to deconvolute or unmix spectra from fluorophores with similar emission peaks, thereby increasing the library of usable fluorophores compared with conventional cytometers with single bandpass filters. These advances maximize the information that can be obtained regarding the diverse leukocyte subpopulations involved in the pulmonary inflammatory response. Second, a unique capability of spectral flow cytometry is that it can use cellular autofluorescence (AF) to aid in identifying cell populations (1). This is particularly useful to pulmonary biologists as the lungs have cell populations with intrinsically high AF properties. Multiple cellular factors (including cell size, granularity, metabolism, and activation), the heterogeneity of immune cell subtypes, and sample processing conditions contribute to AF (2). Although all cells contribute to AF, eosinophils and cells that produce and metabolize surfactants—alveolar type II cells and alveolar macrophages—have the highest AF intensity in the lungs and inflammatory conditions can increase cellular AF (3). Thus, a heterogeneous leukocyte response elicits AF complexity that can pose challenges for analyzing the pulmonary immune response by flow cytometry. In conventional flow, AF is generally viewed as an undesirable source of background that must be extracted, decreasing the sensitivity of fluorophore detection and potentially contributing to the misidentification of cell populations. In contrast, spectral flow cytometry can use cellular AF to define multiple heterogeneous AF signatures, which can be incorporated into a flow panel as discrete signatures, aiding in the resolution and identification of target-specific fluorescent signals.

Using in vivo compartmental analysis facilitates the precise determination of the location and phenotype of leukocytes within the multiple anatomical and functional compartments of the lungs (4). Standard methods of bronchoalveolar lavage followed by lung perfusion and tissue digestion for defining leukocytes in the airways versus the lung interstitium are now recognized to provide limited and often incorrect information regarding pulmonary leukocyte localization. Both bronchoalveolar lavage and lung perfusion provide incomplete recovery and removal of leukocytes within the airways and vasculature, respectively (5–12). It has been demonstrated that perfusion is inadequate to flush the vasculature of the lungs and that many immune cells remain adhered to the endothelium of blood vessels or marginated to the vascular wall. Furthermore, certain lymphocyte subpopulations are preferentially marginated, depending on the agonist and the kinetics of the inflammatory response (5). As the lungs are highly vascularized, these marginated lymphocytes could potentially include large numbers of cells. Similarly, it has been found that bronchoalveolar lavage is inadequate for recovering immune cells from the airways. In particular, it has been shown that increased macrophage activation and adherence in the airways occur in response to lung injury (12, 13). Understanding the biological roles that marginated and adherent leukocytes have in the inflammatory response requires more detailed and precise compartmental analyses of leukocyte trafficking in the lungs.

Thus, we have developed a comprehensive protocol and fluorophore panel combining spectral flow cytometry with in vivo compartmental analysis to investigate leukocyte recruitment into the lungs and mediastinal lymph nodes of control and influenza A virus (IAV)-infected C57BL/6 wild-type mice. Within the lungs, three pulmonary compartments are analyzed: the marginated vasculature (i.e., nonperfusable leukocytes), the lung interstitium, and the airspaces of the lungs (i.e., the lavageable and nonlavageable airways). We incorporate a broad panel of antibodies for immunophenotyping multiple myeloid and lymphocyte populations participating in the inflammatory response. Although previous studies have applied in vivo compartmental analysis to investigate select subsets of leukocytes, here we have established a panel of 20 antibody-fluorophore conjugates that clearly define multiple myeloid and lymphoid cell phenotypes (5, 12, 14). These include B cells, T cells (cytotoxic T, T helper, and γδ T cells), natural killer (NK) cells, macrophages (alveolar and recruited), monocyte-macrophages, monocytes (Ly6C^lo^ and Ly6C^hi^), dendritic cells (CD103^+^ and CD11b^+^), neutrophils, and eosinophils. Employing dual in vivo CD45 labeling of perfused and lavaged lungs offers even more insight regarding the precise localization of adherent leukocytes not only in the vasculature but also in the lung interstitium and the airspaces. In addition, our panel design strategy accounts for AF heterogeneity, by incorporating AF as discrete signatures in our panel, thereby enhancing the panel resolution and increasing the rigor and reproducibility of our results. To our knowledge, the analytical approach for incorporating AF heterogeneity as applied to studies in lungs has not been covered elsewhere. It is a novel contribution of spectral flow cytometry that markedly enhances the quality of experimental data collected from pulmonary tissues.

With this unique combination of methodologies, it was possible to establish an unbiased exploratory approach to investigating the pulmonary immune response to IAV in which no preexisting hypothesis of cellular involvement was needed. Rather, it became possible to investigate multiple cell types, compartments, and time points to identify subclasses of myeloid and lymphoid cells that might have more prominent previously unidentified roles in individual compartments of the lungs depending on the kinetics of the immune response. Furthermore, the overarching goal was to define and detail experimental design and analysis elements supporting rigor and reproducibility in these studies (15, 16). We propose that this protocol also will be of considerable benefit for the detailed understanding of leukocyte trafficking in the lungs in response to other mouse models of lung inflammation and injury.

## METHODS

### Animals

All experiments were approved by the University of Washington Institutional Animal Care and Use Committee (IACUC). Mice were obtained from Jackson Laboratories (Bar Harbor, ME) and bred at the University of Washington. Wild-type 9- to 12-wk-old male C57BL/6J mice were used for all experiments.

### Induction of IAV Pneumonia

Mice were infected with 20 plaque-forming units (PFUs) of mouse-adapted Influenza A/Puerto Rico/8/34 (IAV PR8) in 50 µL of PBS (Thermo Fisher Scientific Waltham, MA) by oropharyngeal aspiration under isoflurane (Patterson Veterinary Supply, Houston, TX) anesthesia (17). A dose of 20 PFU causes severe influenza pneumonia in which mice approach humane euthanasia endpoint criteria but ultimately recover from infection (18). Control mice were instilled with PBS.

### Lipopolysaccharide, Bleomycin, House Dust Mite Administration

Lipopolysaccharide (LPS, *Escherichia coli* O111: B4, List Biological Laboratories, Campbell, CA) was administered by oropharyngeal aspiration under isoflurane anesthesia at a dose of 2.0 mg/kg of mouse body weight in 50 µL of PBS.

Bleomycin (Fresenius Kabi, Bad Homburym, Germany) was administered by oropharyngeal aspiration under isoflurane anesthesia at a dose of 2.0 U/kg of mouse body weight in 50 µL of PBS.

House dust mite (HDM) extract (Greer Laboratories Inc, Lenoir, NC) was administered by oropharyngeal aspiration under isoflurane anesthesia at a dose of 5.0 mg/kg of mouse body weight in 50 µL of PBS. Additional instillations were performed on *days 14, 26, 27*, and *28* after initial instillation.

### Panel Design for Spectral Flow Cytometry

Our strategy was based on recommended workflows for high-dimensional immunophenotyping assays using spectral flow cytometry (19–21). These workflows were developed for use on the Cytek Aurora (Cytek Biosciences, CA) spectral flow cytometer, which we used for our experiments. This panel design strategy included assessment of each tissue for heterogeneous autofluorescent (AF) signatures, which were determined by evaluation of spectra from unstained bronchoalveolar lavage, lung, and lymph node cells from control (PBS instilled) and IAV PR8-infected mice collected at 3, 6, and 9 days after instillation (dpi). Unique AF spectral signatures were identified and designated as fluorescent tags in the SpectroFlo library. These signatures were given high consideration during the assignation of fluorophores to the panel as it is important to avoid selecting fluorophores that share the same peak emission channels or have similar spectral signatures to unique autofluorescence signatures. This panel design also incorporated multiple CD45 antibody-fluorophore conjugates for dual in vivo labeling of the pulmonary intravascular and airspace compartments. We used a fluorophore and antigen optical layout table (Supplemental Table S1) and the Cytek Full Spectrum Viewer Similarity and Complexity matrix (Supplemental Fig. S1) as planning tools to prevent fluorophore spillover-spreading error, reduce panel complexity, and increase the rigor of the panel that was developed. In addition, we titrated all antibody-fluorophore conjugates using cells from 9 dpi IAV PR8-infected lung homogenate tissue to improve the resolution of cell populations in our supervised gating strategy (Supplemental Fig. S2). A comprehensive guide to the panel design strategy is provided in the appendix.

### In Vivo Antibody Labeling and Tissue Harvest

Mice were euthanized at 3, 6, or 9 days after instillation (dpi) with IAV PR8, 3 dpi with LPS, 10 dpi with bleomycin, and 31 dpi with HDM extract. Before euthanasia, mice were anesthetized with isoflurane in an induction chamber. Alexa Fluor 488-conjugated anti-CD45.2 antibody, clone 104 (BioLegend, San Diego, CA), diluted 1:40 (vol:vol) with PBS, in a total volume of 200 µL (2.5 µg per mouse), was injected retro-orbitally to label the intravascular (IV) leukocytes (IVCD45^+^). After 3 min, while keeping the mouse anesthetized with isoflurane delivered by nose cone, the left renal artery was transected, and the mouse was euthanized by exsanguination to reduce the chance that the IVCD45 conjugate would bind nonspecifically to airway or interstitial lung leukocytes. Undesired cross-labeling of lung parenchyma leukocytes with the blood-borne antibody could result from loss of vascular integrity allowing leakage of the intravascular CD45 antibody into the parenchyma. The trachea was cannulated with an 18-gauge angiocath via tracheostomy and BUV615-conjugated anti-CD45 antibody, clone I3/2.3 (BD Biosciences, San Jose, CA) diluted to 1.25 µg/mL with PBS, in a total volume of 1 mL per mouse, was instilled by intratracheal (IT) administration into the lungs (ITCD45). The antibody was allowed to remain in the lungs for 3 min. To reduce the chance of ITCD45 conjugate binding to lung interstitial leukocytes, the airways were immediately lavaged with 1 mL of PBS/0.5 M EDTA two additional times. The thoracic cavity was opened via midline sternotomy and the lungs were perfused by injection of 5 mL of PBS into the right ventricle. Using a dissecting microscope, the largest mediastinal lymph node was removed from the right dorsal aspect of the trachea. The lymph node was placed directly into 0.5 mL of RPMI containing 10% fetal bovine serum (FBS, Thermo Fisher Scientific Waltham, MA) on ice. Lung tissue was removed by blunt dissection from the primary bronchi and placed on ice. Mice that were used for the harvest of unstained tissue did not receive IVCD45 or ITCD45. With these tissue-handling practices, we achieved good compartmentalization of ITCD45^−^ IVCD45^+^, ITCD45^+^ IVCD45^−^, and ITCD45^−^ IVCD45^−^ leukocytes, with minimal (<0.6%) double-positive cells in our experimental model. A higher percentage of co-positive cells might indicate transvascular leakage into airways due to injury caused by the disease process under study or excessive force during the bronchoalveolar lavage or lung perfusion procedures.

### Preparation of Single-Cell Suspension

Cells in the bronchoalveolar lavage (BAL) fluid were pelleted and RBC lysis buffer (Thermo Fisher Scientific Waltham, MA) was applied according to manufacturer instructions. After RBC lysis, BAL cells were pelleted and suspended in Flow Cytometry Straining (FCS) buffer (Thermo Fisher Scientific Waltham, MA). A detailed description of the preparation of reagents used for lung digestion is provided in the appendix. Briefly, lungs were minced using a razor blade and then incubated for 45 min at 37°C in 2-mL RPMI 1640 without phenol red or FBS (Thermo Fisher Scientific Waltham, MA) containing 0.26 U/mL Liberase TM (Sigma-Aldrich, St. Louis, MO) and 10 U/mL recombinant DNase I (Sigma-Aldrich, St. Louis, MO). After digestion, samples were pipetted up and down and then filtered through 70-µm cell strainers (VWR, Brisbane, CA) with RPMI/10% FBS. Lung cells were pelleted and RBC lysis buffer was applied according to manufacturer’s instructions. After RBC lysis, lung cells were pelleted and suspended in FCS buffer. Lymph nodes were transferred onto 70-µm cell strainers and gently mashed with the rubber end of the plunger from a 3-mL syringe. The filters were rinsed with RPMI/10% FBS and then the lymph node cells were pelleted and suspended in FCS buffer. Total live cells from each BAL, lung, and lymph node sample were counted using ViaStain AO/PI staining solution (PerkinElmer Inc, Waltham, MA) and SD100 counting chambers (PerkinElmer Inc, Waltham, MA) on a Cellometer Auto 2000 Cell Counter (Nexcelom, Lawrence, MA).

### In Vitro Antibody Staining

A detailed description of the preparation of reagents and the protocol used for in vitro antibody staining of full-stained samples, single-stained controls, and unstained controls is provided in the appendix. Antibody manufacturer, clone numbers, and titers are available in Supplemental Table S2. All antibodies are commercially available and unstained, single-stain, and fluorescence minus one controls were used to define gating for positive and negative populations. Antibody specificity was not independently validated for antibodies in our panel; however, emphasis was made to select antibody clones recommended from published studies (22–28). Briefly, for full-stained samples, in vitro antibody staining was performed on single-cell suspensions from the BAL, lungs, and lymph nodes of mice that received dual in vivo antibody labeling of IVCD45 and ITCD45. Cells were washed in PBS before being incubated in LIVE/DEAD Fixable Blue Dead Cell Stain (Thermo Fisher Scientific Waltham, MA) for 15 min. This incubation must be performed in a protein-free solution, without FCS, as protein can impact the efficiency of LIVE/DEAD Blue staining. Next, cells were washed with FCS, and brilliant stain buffer was added to each sample. Cells were incubated with Fc Block for 5 min and then incubated with fluorophore-conjugated antibodies for 30 min. Cells were washed with FCS and then incubated with Cytofix (BD Biosciences San Jose, CA) for 15 min. Cells were washed with FCS before being transferred to 5-mL tubes and stored at 4°C in the dark overnight. Flow cytometry was performed the following day.

### Flow Cytometry

**Figure 1.**
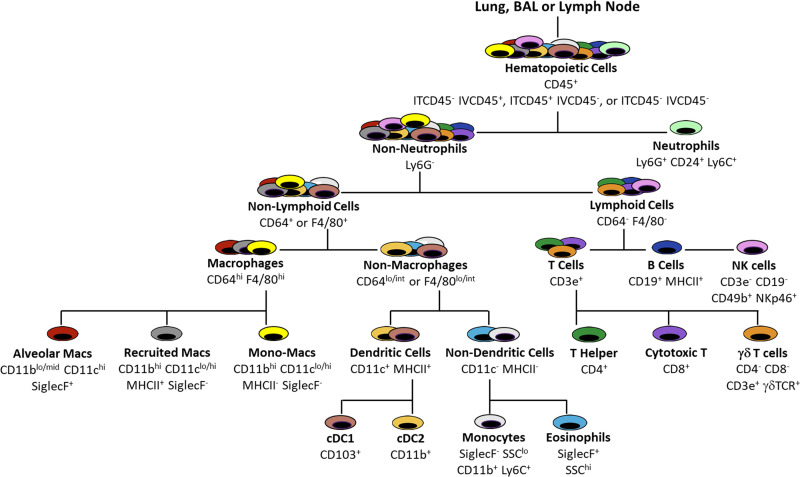
Gating strategy. This spectral flow cytometry panel allows for the characterization of the majority of immune cells involved in the pulmonary immune response to influenza virus including neutrophils, B cells, NK cells, T-helper cells, cytotoxic T cells, γδ T cells, alveolar macrophages, recruited macrophages, monocyte-macrophages, monocytes, CD103+ dendritic cells, CD11b+ dendritic cells, and eosinophils.

A detailed description of the flow cytometry data acquisition is provided in the appendix. Briefly, spectral cytometry was performed using the Aurora cytometer (Cytek Biosciences, Fremont, CA). Our gating strategy is detailed in Fig. 1 and Supplemental Figs. S3, *A–D*, S4, *A–D*, and S5, *A* and *B*. It supports the identification of neutrophils, macrophages, dendritic cells, monocytes, eosinophils, lymphocytes, and natural killer cells. The cell surface markers used were chosen after carefully reviewing the literature, particularly the works of Misharin, McCubbrey, Gibbings, and Tighe and OMIPs -032, -061, and -069 (22–29). All cytometry data were analyzed using SpectroFlo (Cytek Biosciences, CA) or FlowJo (Becton, Dickinson & Company, OR) flow cytometry software. A detailed description of the protocol for spectral unmixing with and without heterogeneous AF signatures is provided in the appendix. t-distributed stochastic neighbor embedding (t-SNE) analysis was initiated downstream of single live CD45^+^ gating on BAL and lung digest cells. Cells were downsampled to 40,000 events and concatenated for t-SNE analysis in FlowJo. Cell subsets defined by our supervised gating strategy were projected onto the t-SNE space to define leukocyte populations in each compartment.

### Statistical Analysis

Statistical analyses and graphs were generated using GraphPad Prism (GraphPad Software, Inc., San Diego, CA). Statistical analyses were performed using ordinary two-way ANOVA with multiple comparisons. Statistical results with a value of *P* < 0.05 were considered statistically significant. All graphs show medians with standard errors.

## RESULTS

### Spectral Flow Cytometry Panel for Compartmental Analysis of the Pulmonary Immune Response

We developed a gating strategy (Fig. 1 and Table 1) and fluorophore panel (Supplemental Table S2) for spectral flow cytometry that is compatible with heterogeneous AF and characterizes B cells, T cells (cytotoxic T, T helper, and γδ T cells), NK cells, macrophages (alveolar and recruited), monocyte-macrophages, monocytes (Ly6C^lo^ and Ly6C^hi^), dendritic cells (CD103^+^ and CD11b^+^), neutrophils, and eosinophils in the pulmonary tissues during influenza infection. Our panel includes dual in vivo CD45 labeling for compartmental analysis of immune cells in the marginated vasculature, lung interstitium, lavageable airway, and nonlavageable airway. In addition, our tissue collection procedures include the collection of the mediastinal lymph nodes for an enhanced understanding of immune trafficking in response to influenza A infection. This approach and protocol leverage spectral flow cytometry to locate, with high precision and resolution, a wide variety of leukocytes during the pulmonary immune response; this combination of approaches is not currently available in published protocols.

**Table 1.** Gating strategy

Tissue Type	Antigen Markers
Marginated vasculature	IVCD45^+^ITCD45^−^CD45^+^ collected from lung homogenate
Lung interstitium	IVCD45^−^ITCD45^−^CD45^+^ collected from lung homogenate
Nonlavageable airways	IVCD45^−^ITCD45^+^CD45^+^ collected from lung homogenate
Lavageable airways	IVCD45^−^ITCD45^+^CD45^+^ collected from bronchoalveolar lavage fluid
Mediastinal lymph nodes	IVCD45^−^CD45^+^ collected from lymph node

Cell Type	Antigen Markers
Neutrophils	Ly6G^+^ CD24^+^ Ly6C^lo/hi^
Lymphoid cells	CD64^−^ F4/80^−^
B cells	CD64^−^ F4/80^−^ CD19^+^ MHCII^+^
T cells	CD64^−^ F4/80^−^ CD3e^+^
Cytotoxic T cells	CD64^−^ F4/80^−^ CD3e^+^ CD8^+^
T-helper cells	CD64^−^ F4/80^−^ CD3e^+^ CD4^+^
γδ T cells	CD64^−^ F4/80^−^ CD3e^+^ CD4^−^ CD8^−^ γδTCR^+^
NK cells	CD64^−^ F4/80^−^ CD3e^−^ CD19^−^ NKp46^+^ CD49b^+^
Non-B Non-T Non-NK cells	CD64^−^ F4/80^−^ CD3e^−^ CD19^−^ NKp46^−^ CD49b^−^
Nonlymphoid cells	CD64^+^ or F4/80^+^
Macrophages	CD64^hi^ F4/80^hi^
Resident alveolar macrophages (Vehicle-instilled mice)	CD64^hi^ F4/80^hi^ CD11b^−^ CD11c^+^ SigF^hi^ Ly6C^mid^ MHCII^−^
Resident alveolar macrophages (Influenza-infected mice)	CD64^hi^ F4/80^hi^ CD11b^mid/hi^ CD11c^+^ SigF^hi^ Ly6C^mid^ MHCII^−^
Recruited macrophages	CD64^hi^ F4/80^hi^ CD11b^+^ CD11c^lo/hi^ SigF^lo^ Ly6C^hi^ MHCII^+^
Monocyte-macrophages	CD64^hi^ F4/80^hi^ CD11b^+^ CD11c^lo/hi^ SigF^lo^ Ly6C^hi^ MHCII^−^
Dendritic cells	CD64^lo/int^ or F4/80^lo/int^ CD11c^+^ MHCII^+^
CD11b^+^ dendritic cells	CD64^lo/int^ or F4/80^lo/int^ CD11c^+^ MHCII^+^ CD11b^+^
CD103^+^ dendritic cells	CD64^lo/int^ or F4/80^lo/int^ CD11c^+^ MHCII^+^ CD103^+^
Monocytes	CD64^lo/int^ or F4/80^lo/int^ CD11b^+^ CD11c^−^ MHCII^−^ SigF^−^ Ly6C^lo/hi^ SSC^lo^
Eosinophils	CD64^lo/int^ or F4/80^lo/int^ CD11c^−^ MHCII^-^ SigF^+^ SSC^hi^

In our gating strategy, after defining single live cells, CD45^+^ leukocytes are segregated based on the expression of ITCD45 and IVCD45. Next, neutrophils are identified as Ly6G^+^ and verified as CD24^+^ with variable expression of Ly6C. From the non-neutrophil gate, lymphoid cells are distinguished from other nonlymphoid cells based on CD64 and F4/80 expression. Lymphoid cells are CD64^−^ F4/80^−^; within the lymphoid gate are CD19^+^ B cells, CD3e^+^ T cells, and CD19^−^ CD3e^−^ non-B non-T cells. B cells are verified as CD19^+^ and MHCII^ +^. The T-cell population is further characterized as CD4^+^ T-helper cells, CD8^+^ cytotoxic T cells, or CD4^−^ CD8^−^ γδ-TCR^+^ γδ T cells. The non-B non-T lymphoid cell population includes NK cells, as identified with NKp46 and CD49b. Nonlymphoid cells are identified from those that express CD64 or F4/80; within the nonlymphoid gate are monocytes, macrophages, dendritic cells, and eosinophils. CD64^hi^ F4/80^hi^ cells include resident alveolar macrophages (AMs) that are CD11b^−^ CD11c^+^ in control animals or CD11b^mid/hi^ CD11c^+^ in PR8-infected animals; AMs are verified as SiglecF^+^ Ly6C^mid^ MHCII^−^. CD64^hi^ F4/80^hi^ cells also include a CD11b^+^ CD11c^lo/hi^ subset, which can be further divided into MHCII^−^ and MHCII^+^ cells. The nomenclature for macrophage phenotypes that are present in the lung during inflammation and injury is inconsistent in the literature. We term CD64^hi^ F4/80^hi^ CD11b^+^ CD11c^lo/hi^ MHCII^+^ cells as “recruited macrophages” (RMs) (30). However, these cells are phenotypically consistent with what other investigators have termed “interstitial macrophages” and “monocyte-derived macrophages” (31, 32). In addition, we term CD64^hi^ F4/80^hi^ CD11b^+^ CD11c^lo/hi^ MHCII^−^ cells as “monocyte-macrophages” and verify that they are SiglecF^−^ Ly6C^hi^ (33). Cells that are positive for either, but not both, CD64 or F4/80 include CD11c^+^ MHCII^+^ dendritic cells, which are further identified as CD11b^+^ or CD103^+^ dendritic cell subpopulations. Nondendritic cells include SiglecF^−^ SSC-A^lo^ monocytes, which are verified as CD11b^+^ with both Ly6C^lo^ and Ly6C^hi^ subsets, and SiglecF^+^ SSC-A^hi^ eosinophils, which are verified to be Ly6G^−^ CD11c^−^ MHCII^−^.

### Identification of Autofluorescence Signatures

We leveraged the capability of spectral flow cytometry to manage autofluorescence (AF) signatures effectively by first identifying AF from raw spectral data of unstained lymph node, BAL, and lung cells from control (PBS) and IAV-infected mice at 3, 6, and 9 dpi. In contrast to conventional flow cytometers, which only record a portion of the light spectrum, spectral cytometers record a full spectrum of signal from inherent cellular AF and fluorophores (20). The range of the full spectrum is determined by the instrument configuration and is dependent on the number of available lasers, dispersion of light source by the prism, and method of light detection (34). The instrument used in our study, the Cytek Aurora 5 Laser 16 ultraviolet - 16 violet - 14 blue - 10 yellow-green - 8 red (16UV-16V-14B-10YG-8R) system, has 64 fluorescence detectors, which capture wavelength of light from 380 to 800 nm. The intensity (brightness, *y*-axis) of autofluorescence captured by each detector (*x*-axis) is plotted to create an AF spectra for each unstained tissue type. The autofluorescence plots are displayed as a heat map with the red-to-orange portion of the band representing intensity measure counts occurring at high frequency, the yellow-to-green portion of the band representing intensity measure counts occurring at intermediate frequency, and the cyan-to-blue portion of the band representing intensity measure counts that occur rarely. Heterogeneous AF signatures are signatures that contain more than one distinct AF signature and will often present with multiple red-to-yellow bands or poorly defined red bands within a signal detector.

**Figure 2.**
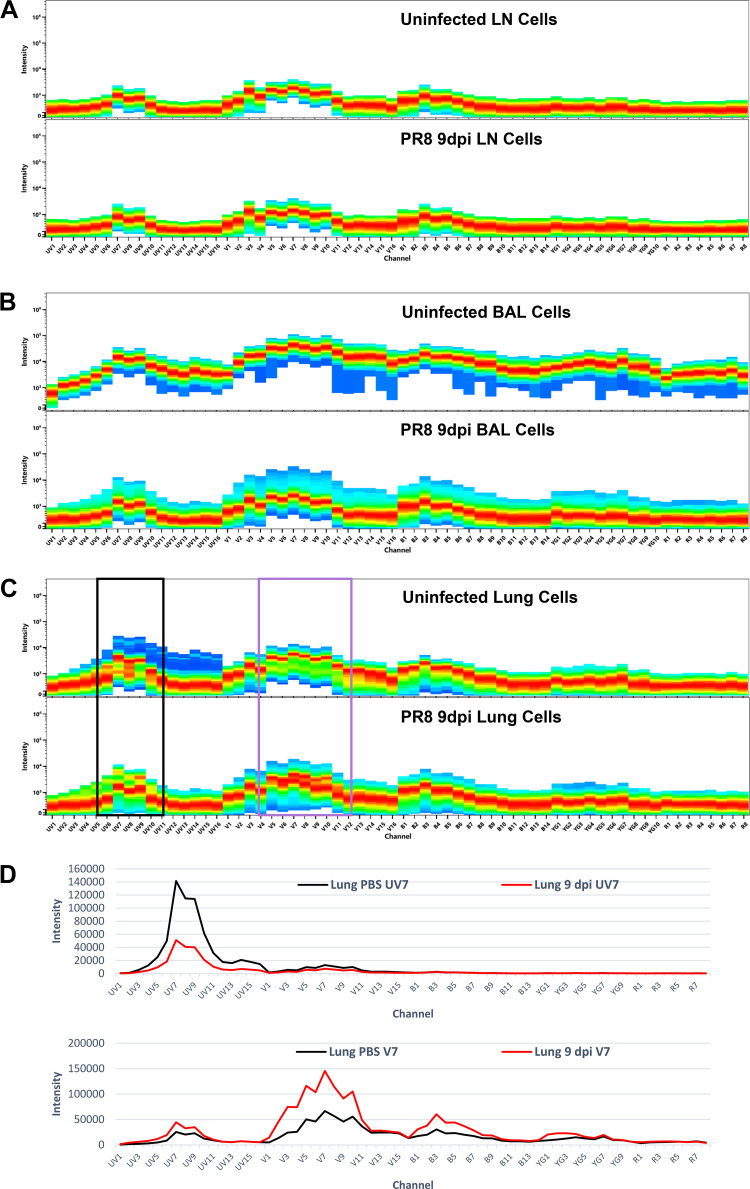
Spectral signatures of unstained lymph node, alveolar airway, and lung cells from PBS control uninfected versus 9 dpi PR8-infected mice. Representative spectra are shown for cells from lymph node (*A*), BAL (*B*), and lungs (*C*) from male mice that were uninfected, or infected with 20 PFU IAV PR8 and euthanized at 9 dpi. All samples were processed for removal of debris and aggregates with SpectroFlo software. *A* and *B*: spectra for lymph node and BAL cells from both uninfected and 9 dpi PR8 mice reflect relatively homogeneous AF, as indicated by a predominant population with a narrow range of intensity (red band) across the spectrum for uninfected and 9 dpi PR8 mice. *C*: spectra for lung cells from both uninfected and 9 dpi PR8 mice reflect heterogeneous AF, as indicated by multiple prominent populations (ranging in intensity from red to yellow) particularly in the ultraviolet (black box) and violet channels (purple box). *D*: spectral overlays of the UV7 AF signature and V7 AF signature identifies the distinct AF signatures present in control and PR8-infected lungs. AF, autofluorescence; BAL, bronchoalveolar lavage.

In the lymph nodes of a control mouse, the spectral signature shows low fluorescence intensity with little variation across the emission spectrum, indicating high spectral homogeneity of cell populations within this tissue and low AF complexity (Fig. 2*A*). In the BAL of a control mouse the spectral signature shows high fluorescence intensity of a fairly homogenous cell population across the emission spectrum (Fig. 2*B*). In lung cells from a control mouse, the spectral signature reveals a broader range of intensity across the spectrum, with the primary (red) band exhibiting lower fluorescence intensity than for BAL cells. In addition, the primary band is notably broad in the ultraviolet and violet channels and discontinuous, with multiple prominent bands (red-orange or yellow) in the mid-UV channels and in the mid-V channels, indicating AF complexity due to the presence of multiple spectrally distinct cell populations (Fig. 2*C*).

On evaluating unstained BAL and lung digest samples, we find that AF signatures vary with the kinetics of the immune response (Supplemental Figs. S6 and S7). BAL cells have homogenous AF signatures that shift to lower fluorescence intensity over the course of infection (Supplemental Fig. S6). BAL cells have a broad range of intensity of AF in the ultraviolet and violet channels at 6 dpi, reflecting heterogeneous AF, that narrows by 9 dpi (Fig. 2*B* and Supplemental Fig. S6). Lung cells display distinct spectral characteristics within the ultraviolet and violet channels at each of the time points examined (Fig. 2*C* and Supplemental Fig. S7). These shifts reflect the dynamic nature of recruitment and maturation of different leukocyte populations with heterogeneous AF over the time course of the inflammatory response. In contrast, the spectral signatures of lymph node cells remained similar over the course of infection with low indication of spectrally distinct cell populations or AF heterogeneity (Fig. 2*A* and Supplemental Fig. S8).

Using the approach for managing heterogeneous AF, described in detail by Ferrer-Font et al. (35), two distinct high-intensity AF signatures, with peaks in the UV7 and V7 channels, were identified in control uninfected lungs (Fig. 2*D*). These signatures change subtly over the course of infection. Overlays of the UV7 signatures from control, 3, 6, and 9 dpi lungs reveal differences in intensity over the course of infection, particularly for the 9 dpi signature, which is reduced in intensity in the ultraviolet region of the spectrum compared with other UV7 signatures, whereas the 9 dpi V7 signature has increased intensity in the violet channels compared with other V7 AF signatures (Fig. 2*D* and Supplemental Fig. S7*B*). Correct identification of AF spectra that are of high intensity is particularly important for unmixing as these signatures act as single stain controls once entered into the SpectroFlo library. Erroneously using the low-intensity 9 dpi UV7 AF spectra for unmixing data from control, 3, or 6 dpi results in significant unmixing errors. This is similarly true if low-intensity V7 AF spectra from control, 3, or 6 dpi are used to unmix data from 9 dpi. These findings emphasize the importance of identifying AF spectra at each time point investigated in addition to investigating AF spectra for control and infected mice.

Notably, the spectral characteristics of lung tissues change with not only time but also the inflammation model. We have performed studies to evaluate AF in other murine pulmonary inflammation models, including the response to lipopolysaccharide from *E. coli*, bleomycin, and house dust mite extract from *Dermatophagoides pteronyssinus* (Supplemental Fig. S9). The spectral characteristics of unstained lungs from these models are distinct from each other and those observed in the IAV PR8 model (Fig. 2*C* and Supplemental Fig. S7). The house dust mite model is particularly complicated, with three unique AF signatures identified (Supplemental Fig. S9*B*).

### Comparison of AF Management Strategies

**Figure 3.**
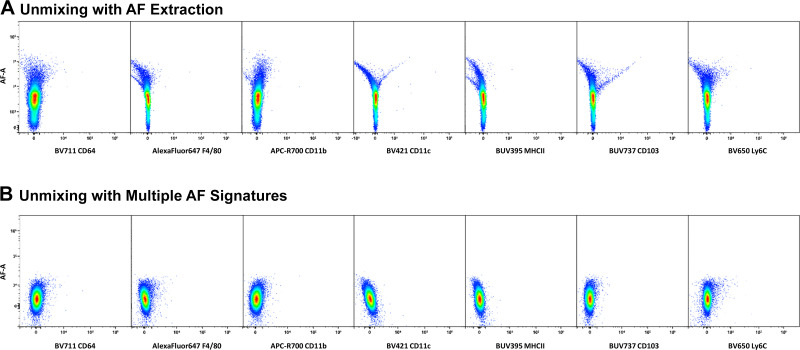
Comparison of AF management strategies. The two strategies of unmixing with AF extraction versus multiple AF signatures were assessed for unmixing errors using unstained lungs of a 9 dpi PR8 mouse. Select N × 1 plots are shown here for fluorophores conjugated to antibodies for phenotyping macrophages, dendritic cells, and monocytes. *A* and *B*: errors were observed when unmixed with AF extraction (*A*), as indicated by both positive and negative skewing of populations, whereas well-shaped round populations were achieved when unmixed with multiple AF signatures (*B*). Certain fluorophores were more negatively impacted by AF (e.g., AlexaFluor 647, BV421, BUV395, BUV737, and BV650) than others. AF, autofluorescence.

We further examined the ability of the deconvolution algorithm to clearly resolve fluorophore signatures in our complex panel by evaluating processed data for unstained lung leukocytes that were unmixed by two strategies—with standard AF extraction or with the inclusion of multiple AF signatures. For this, we visualized N × 1 plots of AF versus every other fluorophore and looked for unmixing errors. In both strategies, the “AF as a tag” feature in the SpectroFlo software was used. Without the inclusion of distinct AF signatures, errors were observed with many of the fluorophores in our panel, as evidenced by both positive and negative skewing of populations as opposed to being symmetrically centered around zero (Fig. 3*A*). Here, we show fluorophores conjugated to antibodies primarily for phenotyping nonlymphoid cells (macrophages, dendritic cells and monocytes), but errors were observed with several fluorophores conjugated to antibodies for lymphoid cells as well (not shown). These unmixing errors indicate improper resolution of positive and negative populations and can cause fluorescence from endogenous AF to be improperly assigned to other fluorophores. Including our two unique AF signatures achieves well-shaped clean and round populations centered around zero, which should markedly improve the assignation of fluorescence signatures to their correct fluorophores (Fig. 3*B*).

### Compartmentalization of the Pulmonary Inflammatory Response Using Dual In Vivo CD45 Labeling

**Figure 4.**
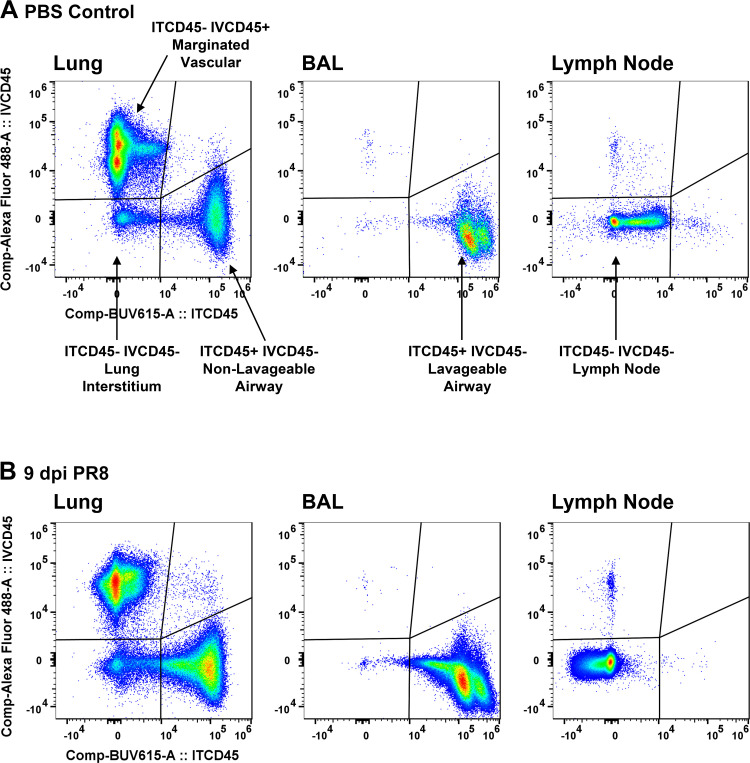
Compartmentalization of the pulmonary inflammatory response. Leukocytes were labeled in vivo with intravascular AlexaFluor 488 anti-CD45 (IVCD45), intratracheal BUV615 anti-CD45 (ITCD45), and ex vivo with PerCP anti-CD45 (CD45). Single live CD45^+^ cells were evaluated for IVCD45 and ITCD45 staining in lungs, BAL, and lymph nodes of representative PBS control (*A*) and 9 dpi male PR8-infected mice (*B*). BAL, bronchoalveolar lavage.

The compartmental localization of leukocytes in the pulmonary inflammatory response was defined by labeling leukocytes in vivo, first with intravenous AlexaFluor488-conjugated anti-CD45 (IVCD45) and second with intratracheal BUV615-conjugated anti-CD45 (ITCD45). After tissue collection and preparation of single-cell suspensions, leukocytes were labeled in vitro with a full-stain fluorophore-antibody master mix including PerCP anti-CD45 (CD45) and analyzed by flow cytometry. After gating on single live CD45^+^ leukocytes, we used quadrant gating to evaluate events for IVCD45 or ITCD45 staining (Fig. 4 and Table 2). The four quadrants reflect ITCD45^−^ IVCD45^+^ (top left), ITCD45^+^ IVCD45^+^ (top right), ITCD45^+^ IVCD45^−^ (bottom right), and ITCD45^−^ IVCD45^−^ (bottom left) events. In BAL of both control and PR8-infected mice, >98% of CD45^+^ cells are ITCD45^+^ IVCD45^−^ and represent lavageable leukocytes; we call these lavageable leukocytes; <2% of BAL cells fall into the other quadrants. In the lungs of control mice, 73.73 ± 1.49% of CD45^+^ cells are ITCD45^-^ IVCD45^+^ and represent vascular cells that remain in the lung after IV perfusion; these are marginated vascular leukocytes. CD45^+^ (19.43 ± 1.66%) cells are ITCD45^+^ IVCD45^−^ and represent alveolar cells that remain in the lungs after three lavages with PBS/0.5 M EDTA; these are nonlavageable leukocytes. Only 6.61 ± 1.02% of CD45^+^ cells in control mice are double-negative for the IT and IV labels; these are true lung interstitial leukocytes. In the lungs of PR8-infected mice, the distribution of leukocytes in the three lung compartments changes, with 23.21 ± 2.27% in the marginated vascular, 48.39 ± 1.23% in the nonlavageable airway, and 27.60 ± 2.75% in the interstitial compartment. In the lungs of both PBS and PR8 mice, <0.6% of cells are double-positive for the IT and IV labels, indicating negligible vascular leakage into airway compartments. In the lymph nodes of both control and PR8-infected mice, ∼99% of CD45^+^ cells are double-negative for both ITCD45 and IVCD45 and represent lymph node leukocytes. Thus, this analysis reflects good segregation of leukocytes in the four pulmonary compartments. It also reveals that the majority of airway leukocytes are resistant to three lavages at 9 dpi with IAV PR8.

**Table 2.** Compartmentalization of leukocytes

	% Single Live CD45^+^ Cells			
	ITCD45^−^ IVCD45^+^	ITCD45^+^ IVCD45^+^	ITCD45^+^ IVCD45^−^	ITCD45^−^ IVCD45^−^
PBS BALs^	0.54 ± 0.17	0.08 ± 0.02	98.44 ± 0.36	0.94 ± 0.25
PR8 BALs^	0.04 ± 0.03	0.01 ± 0.00	99.44 ± 0.11	0.51 ± 0.12
PBS Lungs#	73.73 ± 1.49	0.24 ± 0.10	19.43 ± 1.66	6.61 ± 1.02
PR8 Lungs#	23.21 ± 2.27	0.56 ± 0.04	48.39 ± 1.23	27.60 ± 2.75
PBS LNs^	0.45 ± 0.19	0.00 ± 0.00	0.25 ± 0.14	98.78 ± 0.18
PR8 LNs^	0.10 ± 0.08	0.00 ± 0.00	0.06 ± 0.03	99.72 ± 0.08

Values are means ± SE. Data reflect *n* = 5 male PBS mice and *n* = 6 male PR8 mice. Quadrant gating was used to evaluate single live CD45^+^ cells for ITCD45 or IVCD45 staining in BAL, lungs, and lymph nodes (LN) of PBS control or 9 dpi PR8-infected mice. ITCD45^−^ IVCD45^+^ reflect marginated vascular cells in the lungs; ITCD45^+^ IVCD45^−^ reflect lavageable and nonlavageable airway cells in the BAL and lungs, respectively; ITCD45^−^ IVCD45^−^ reflect cells in the lung interstitium or lymph nodes. ITCD45^+^ IVCD45^+^ reflects leakage from the vasculature into the airways and was negligible. AF, autofluorescence; BAL, bronchoalveolar lavage.

^BAL and LN samples did not have heterogeneous AF and were unmixed with AF extraction.

#Lung samples had heterogeneous AF and were unmixed with multiple AF signatures.

### Supervised Gating on Pulmonary Leukocytes of PR8-Infected Mice

Representative supervised gating trees on leukocytes in the marginated vasculature, lung interstitium, nonlavageable airways, lavageable airways, and mediastinal lymph nodes are shown in Supplemental Figs. S3, *A–D*, 4, *A–D*, and 5, *A* and *B*. By applying our gating strategy to each compartment in PR8-infected mice, we immunophenotype 94.27 ± 0.26% of all leukocytes in the marginated vasculature, 90.51 ± 0.38% in the lung interstitium, 99.63 ± 0.25% in the airways, and 96.6 ± 0.25% in the lymph nodes. The largest population of undefinable cells (3.75 ± 0.26%) are CD45^+^ ITCD45^−^ IVCD45^−^ Ly6G^−^ CD64^−^ F480^−^ CD19^−^ CD3^−^ NKp46^−^ CD49b^−^, which are lymphoid cells that are non-B non-T non-NK cells within the lung interstitial compartment.

**Figure 5.**
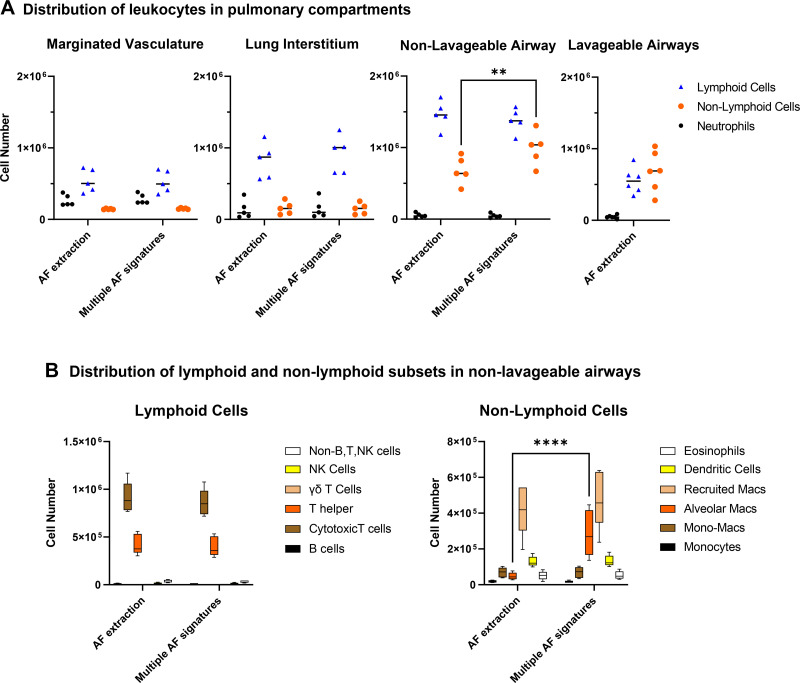
Compartmental localization of leukocytes. Unmixing strategies with AF extraction or with multiple AF signatures were compared for their ability to quantify neutrophils, lymphoid cells, and nonlymphoid cells in four pulmonary compartments (*A*) and lymphoid and nonlymphoid subpopulations in the nonlavageable airway compartment (*B*) of 9 dpi PR8-infected mice (data reflect *n* = 6 male mice; means ± SE; **P < 0.01 and ****P < 0.0001). AF, autofluorescence.

The distribution of leukocyte subpopulations across the multiple pulmonary compartments is shown in Fig. 5. This analysis also further examines unmixing strategy options. Several findings are observed when incorporating multiple AF signatures. First, 60.5 ± 12.4% of all neutrophils remain marginated in the pulmonary vasculature rather than migrating into other compartments, and 4.9 ± 2.1% remain nonlavageable. Second, 18.7 ± 1.7% of lymphoid cells and 17.1 ± 2.6% of nonlymphoid (not including neutrophils) are marginated in the vasculature. Third, the majority of leukocytes, 50.5 ± 1.3% of lymphoid and 67.5 ± 1.7% of nonlymphoid cells, are nonlavageable. Fourth, 44.5% more (*P* < 0.01) nonlymphoid cells are identified in the nonlavageable airway compartment when unmixed with the inclusion of two AF signatures than without (Fig. 5*A*). Simultaneously, there are fewer numbers of unidentified cells that do not fall into any of our immune cell phenotypes when unmixing with inclusion of AF signatures than without (not shown). Finally, incorporating AF complexity significantly improves the quantification of alveolar macrophages in the nonlavageable airways, revealing sixfold more (*P* < 0.0001) cells compared with unmixing with standard AF extraction (Fig. 5*B*). This reflects the improved resolution and assignation of fluorescence as shown in Fig. 3. Together, these findings validate the importance of defining the compartmentalization of the pulmonary immune response. Without a dual in vivo CD45-labeling approach, immune cells within the marginated vasculature and nonlavageable airspaces would be incorrectly pooled with lung interstitial leukocytes. In addition, without mitigation of AF complications, highly autofluorescent alveolar macrophages would be underquantified and their involvement in the pulmonary inflammatory response would be misinterpreted.

### Unsupervised Gating on Pulmonary Leukocytes of PR8-Infected Mice

**Figure 6.**
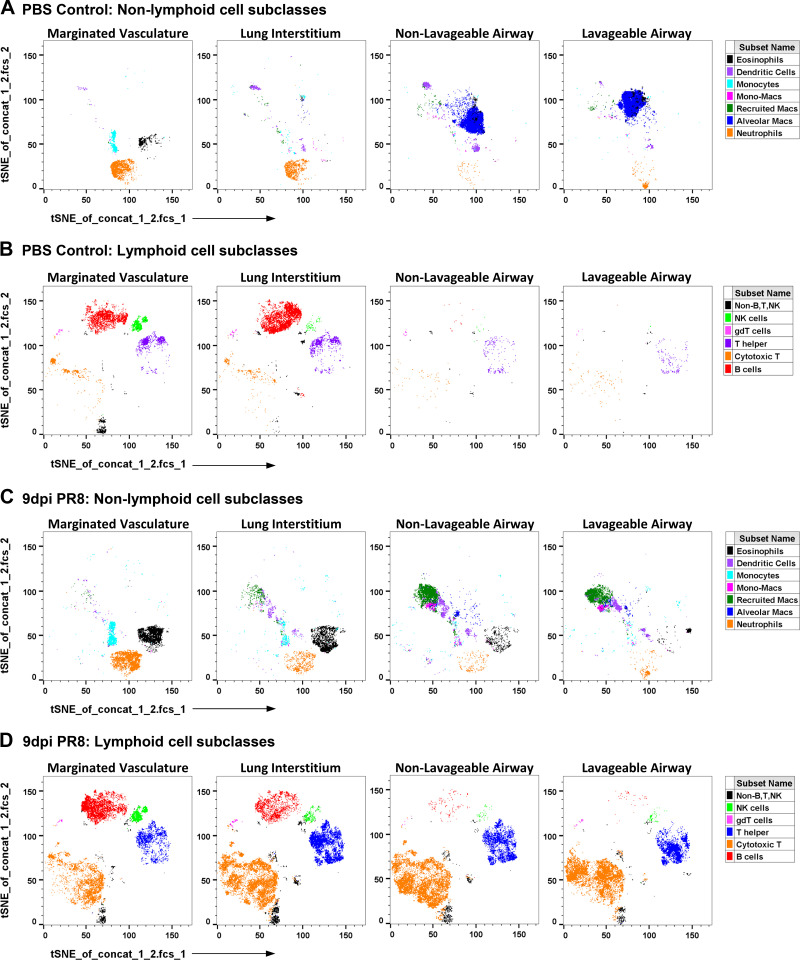
Unsupervised gating. t-SNE analysis was initiated downstream of singlet and viability gating on 40,000 lung digest cells from representative PBS control (*A* and *B*) and 9 dpi male PR8-infected mice (*C* and *D*). Cell subsets defined by the supervised manual gating strategy were projected into the t-SNE space using specific colors to define nonlymphoid and lymphoid cell populations in the marginated vasculature, the lung interstitium, the nonlavageable airway, and the lavageable airway.

We performed t-SNE analysis to aid in visualizing and localizing immune cells within distinct pulmonary compartments of PBS control and PR8-infected mice (Fig. 6). Nonlymphoid cells in PBS control lungs include primarily neutrophils, monocytes, and eosinophils in the marginated vasculature, neutrophils in the lung interstitium, and alveolar macrophages in the nonlavageable and lavageable airways (Fig. 6*A*). Lymphoid cells in PBS control lungs include primarily B, T helper, and NK cells in the marginated vasculature and lung interstitium, with few cells in the airways (Fig. 6*B*). As previously reported, lymphoid cells can be difficult to perfuse and all subclasses are seen in the marginated vascular compartment of both PBS control (Fig. 6*B*) and PR8-infected lungs (Fig. 6*D*) (5). Here, we demonstrate that neutrophils, monocytes, and eosinophils also remain in the vasculature after perfusion in both PBS control (Fig. 6*A*) and PR8-infected lungs (Fig. 6*C*). This analysis also reflects that individual nonlymphoid subpopulations are distinctly localized within the different pulmonary compartments. Alveolar macrophages are the predominant leukocyte in the airways of PBS control mice (Fig. 6*A*), but are a minor component within the airways of 9 dpi PR8-infected mice (Fig. 6*C*). Although monocytes are abundant in the marginated vasculature, they are a minor component of the lung interstitium and not found in the airways of both PBS control (Fig. 6*A*) and PR8-infected lungs (Fig. 6*C*). Conversely, mono-macrophages, recruited macrophages, and dendritic cells are identified in the airways but not in the marginated vasculature of PR8-infected lungs (Fig. 6*C*). This distribution reflects differentiation and maturation of monocytic cells as they are recruited from the circulation into the airways in response to oropharyngeal PR8. In contrast, most lymphoid cell subpopulations, except for B cells, are relatively uniformly distributed across the lung. This t-SNE analysis demonstrates a high correlation with most populations in our supervised gating strategy and confirms many observations from that strategy. It also provides visualization which reflects the dynamic nature of leukocyte migration, recruitment, and activation from the circulation across the lung parenchyma and into the airways in response to influenza infection.

## DISCUSSION

In summary, we present a complete protocol, incorporating spectral flow cytometry with in vivo compartmental analysis, for the precise localization of multiple lymphoid and nonlymphoid cell populations participating in the pulmonary immune response. Here, we apply this methodology to studies in wild-type C57/BL6 mice that were uninfected or 9 dpi with IAV/PR8. Key aspects of this protocol are that it *1*) describes changes in the AF characteristics of control and PR8-infected lungs; *2*) incorporates a meticulously assembled broad panel of 20 antibody-fluorophore conjugates that are compatible with these AF characteristics and spectral flow cytometry; *3*) validates that incorporating multiple heterogeneous AF signatures from the lungs improves the resolution and identification of fluorescence signals, which is of particular importance when alveolar macrophages are a component of the immune response; *4*) establishes a robust gating strategy for identification of B cells, T cells (cytotoxic T, T helper, and γδ T cells), NK cells, macrophages (alveolar, recruited and monocyte-macrophages), monocytes (Ly6C^lo^ and Ly6C^hi^), dendritic cells (CD103^+^ and CD11b^+^), neutrophils, and eosinophils; *5*) combines a dual in vivo CD45-labeling protocol with in vitro CD45 labeling to facilitate identification of these immune cell populations in four pulmonary compartments; *6*) implements both informed gating and dimensionality reduction algorithms to visualize the recruitment and migration of leukocytes from the vasculature, across the lung interstitium and into the alveolar airways; and *7*) finds that the majority of immune cells are localized within the nonlavageable airways in this model at 9 dpi with IAV PR8.

In developing this protocol, we gave heavy consideration to the best practices outlined in the American Thoracic Society Workshop Report, whose intent was to improve the rigor and reproducibility of flow cytometry experiments related to studies in the lungs, as well to broad mandates to improve the rigor and reproducibility of biomedical research (15, 16, 29). The abilities of spectral flow cytometry are particularly well-suited for these goals as it mitigates AF interference and it uses AF characteristics to significantly improve resolution and identification of leukocyte populations. Combined with in vivo compartmental analysis, we can describe >99% of the cellular immune response within the nonlavageable airways, a compartment that has not previously been quantitatively described with precision. We find these procedures to yield high-quality data with easily resolvable cell populations and highly consistent quantitative results between multiple users and over multiple experiments. The clarity of resolution gained by including AF considerations enhances the rigor of unsupervised analyses.

Previous studies also have used in vivo compartmental approaches to better define the localization of pulmonary immune cells. Anderson et. al. (14) used intravascular labeling to demonstrate that up to 97% of CD8+ T cells thought to be located within the lungs of perfused LCMV-infected mice were not in the tissue, but remained adherent in the vasculature. Patel et. al. (12) used intravascular and intratracheal labeling to characterize myeloid cells in the nonlavaged lungs of naïve mice and those with acid inspiration-induced injury. Although they were able to segregate vascular and alveolar cells from those within the lung interstitium, they did not identify those alveolar cells that were nonlavageable from the airspaces of the lungs. Leukocytes that are marginated and nonlavageable, whether in the vasculature or in the airway lumen, warrant more investigation. They could be transitory and poised to leave or they could be restricted from exiting these highly specialized compartments. The precise immunophenotyping and quantification of these populations could aid in understanding the biological roles that these cells have and could inform future therapeutic approaches for pulmonary inflammation.

The protocol presented here enables precise quantitative tracking of lymphoid and nonlymphoid leukocytes in four compartments within perfused and lavaged lungs: the marginated vasculature, the lung interstitium, the lavageable airspaces, and the nonlavageable airspaces. In our model of IAV PR8 at 9 dpi, we show that the majority of pulmonary B cells and NK cells, as well as large populations of CD4^+^ T-helper cells and CD8^+^ cytotoxic T cells, remain adherent in the vasculature after perfusion. Although T-helper cells were distributed across these four compartments, the majority were localized to the nonlavageable airspace compartment, making this location the site with the greatest number of lymphoid cells. We also show that the majority of Ly6C^hi^ monocytes as well as large populations of neutrophils and eosinophils remain adherent in the vasculature, whereas the majority of Ly6C^mid^ monocytes, recruited macrophages, and dendritic cells (primarily CD11b^+^) are adherent in the airway lumen. A small population of alveolar macrophages also are localized in both the lavageable and nonlavageable airspaces. In short, the previously unquantified nonlavageable airspace is the site of the largest number of lymphoid and nonlymphoid cells within the lungs at 9 dpi with IAV/PR8. Thus, by including bronchoalveolar lavage in this protocol, it becomes possible to identify immune cells within the nonlavageable airspace. This approach could potentially offer insight into functionally different subsets of immune cells and could be an area of future study. Inclusion of lavage offers two additional advantages. First, by rapidly diluting and removing the ITCD45 conjugate, lavage reduces the opportunity for cross-labeling of lung interstitial leukocytes. Second, additional valuable information can be obtained from the cell-free BAL fluid, including analytes such as total protein, immunoglobulins, and cytokines. Although some of this information might be more accurately obtained by performing lavage in separate animals, the inclusion of lavage in this protocol allows for more information to be obtained from the same animals, thereby reducing the number of animals in a study. One caveat to our approach is that it used three lavages. If additional lavages were performed, one would expect a decrease in the nonlavageable cells and an increase in the lavageable cells with each additional lavage. Limitations of the work presented are that histological data were not obtained to confirm the specific localization of different cell types and that these experiments were only performed in male mice. Future experiments using immunofluorescent histochemistry will be important for validating our findings. Modifications to this protocol might be required for studies in female mice, as C57BL6/J mice have sex-based differences in susceptibility to IAV PR8 (18).

It is notable that this analysis shows that monocytes in the marginated vasculature of both control and IAV PR8-infected mice include both Ly6C^hi^ and Ly6C^lo^ populations, whereas Ly6C^hi^ monocytes are the majority in the airways of PR8-infected mice (Supplemental Figs. S3 and S4). This is consistent with the characterization of “classical” and “non-classical” monocytes, with shifting Ly6C expression as they transit from the circulation and are recruited into the airways during pulmonary inflammation (36, 37). It is also notable that at 9 dpi, the airspaces contain relatively few alveolar macrophages compared with recruited macrophages, and that the majority of alveolar macrophages are localized to the nonlavageable airway. This contributes to high heterogeneous AF in the lungs, but not in the BAL. In contrast, though not shown here, at the earlier time points of 3 and 6 dpi with PR8, we found considerably more alveolar macrophages in the lavageable airways which contribute to heterogeneous AF. Thus, while at 9 dpi, it was not necessary to define and incorporate discrete AF signatures in the BAL compartment, it was necessary to do so at the earlier time points (not shown). These findings are consistent with the understanding of macrophage turnover during influenza infection (38–40). Furthermore, these findings emphasize the changing nature of AF in different pulmonary compartments over the time course of the immune response.

We also observe from t-SNE analysis a high eosinophil content within the lungs of IAV PR8-infected mice at 9 dpi, with eosinophils constituting 33.0 and 58.3% of all nonlymphoid cells within the marginated vasculature and lung interstitium, respectively. This population is SiglecF^+^ SSC-A^lo^ and are back-gated to confirm that they do not express Ly6G, CD11c, or MHCII to validate their identity as eosinophils rather than neutrophils, alveolar macrophages, recruited macrophages, dendritic cells, or monocytes. Although eosinophils are well-recognized to be modulators of adaptive immune responses and are released from the bone marrow following exposure to allergens or intestinal parasites, their role(s) in host defenses against respiratory viruses is (are) less well-known (41–44). With respect to IAV, it has been described that eosinophils have very low presence in the airways during early time points of infection but increasing numbers that correlate with T-cell recruitment into the lungs, and, more recently, that eosinophils modify the respiratory barrier during IAV infection, helping to neutralize the virus and protect the airways (45, 46). Our observations are consistent with these novel findings and suggest that the capabilities of the protocol described here would be of considerable utility in further studies investigating the dynamics of eosinophil recruitment and migration in response to IAV and other respiratory viruses. These unexpected findings also provide evidence for the utility of taking an unbiased approach to measure changes in leukocyte populations in pulmonary research.

Less than 5% of lung leukocytes cannot be immunophenotyped using the strategies defined here. These are non-B non-T non-NK lymphoid cells and are most prevalent within the lung interstitial compartment. Employing the t-SNE algorithm, these cells are seen to cluster within the two-dimensional space located adjacent to T cells. These could reflect shortcomings in our gating strategy or perhaps lineage negative innate lymphoid cells (ILCs) that could be investigated with additional antibodies (47). Although the panel presented here includes 20 antibody-fluorophore conjugates, it has a low overall complexity index and is amenable to substitution or expansion for investigations of additional leukocyte populations.

As emphasized in our fluorophore panel design strategy, it is essential to assess AF complexity within each pulmonary tissue as part of the experimental design process; knowing the peak emission channels of AF signatures is necessary to ensure compatibility with fluorophores in the in vitro staining panel. We demonstrate that the intensity and signature of heterogeneous AF can change over the course of the immune response to lung inflammation and injury. Therefore, it is important to assess AF complexity for each model of lung inflammation and time point of interest. We found that the house dust mite model had many AF signatures (Supplemental Fig. S9*B*) and this is likely due to high eosinophil recruitment and the inherent highly autofluorescent properties of the chitin structural component within house dust mite extracts (48).

In addition, day-to-day variations in tissue processing can affect AF, therefore it is necessary to include an additional mouse within each experiment for the collection of unstained controls for BAL, lung, and lymph node cells to determine AF signatures unique to each experiment. Although these procedures require considerable effort for optimization and set-up, the clarity of resolution that is gained enables the acquisition of a large amount of high-quality data that could improve efficiency and reduce the numbers of mice required to assess meaningful differences between groups. It has been suggested that apparent inconsistencies in the literature regarding the response of alveolar macrophages to influenza infection might be understood by identifying mouse strain-dependent differences (49). Our results demonstrate that another important consideration for the accurate quantification of alveolar macrophages is the identification of heterogeneous AF signatures, which must be given due consideration to generate rigorous and reproducible flow cytometry data as shown in Fig. 5*B*.

It is expected that different cell populations will be enumerated in different lung compartments during the innate versus adaptive versus resolution and repair phases of the immune response, depending on the disease model under study. During influenza infection, innate effector cells include NK cells, neutrophils, monocytes, macrophages, and dendritic cells. Dendritic cells act as a bridge between innate cellular responses and adaptive cellular responses, which are dominated by T and B lymphocyte subpopulations (50). Thus, this panel was carefully designed to enable flexibility for incorporating cell populations from both the innate and adaptive immune responses and for expansion to include additional fluorophore-antibody conjugates and to incorporate multiple AF signatures. This flexibility will accommodate deeper probing into leukocyte subpopulations of interest (e.g., M1 vs. M2 macrophages, or subsets of activated dendritic cells), extension to acquired immunity (e.g., T regulatory or T memory cells), or application to different inflammatory models in which additional leukocyte populations might be of interest (e.g., basophils and mast cells in allergic airway inflammation). Although not included as part of this protocol, it would be feasible to collect whole blood at time of euthanasia (e.g., by cardiac puncture) and apply the procedures described here for a more thorough characterization of circulating immune cells.

This protocol employs many strategies to advance the rigor and reproducibility of pulmonary research. With regard to rigor, our unbiased exploratory approach provides the opportunity to uncover novel insights about leukocyte populations that may be missed on more focused flow cytometry panels. In combination with dual in vivo CD45 labeling, this protocol allows for the precise characterization and localization of the cellular participants in the immune response. This is crucial to understanding the fundamental processes underlying pulmonary inflammation and for designing therapeutic approaches that target the aberrant leukocyte recruitment in pathological situations. The panel design strategy described in the appendix is essential for the success and reproducibility of this work. We adhere to a high standard of practice for panel set-up, optimization, and management of autofluorescence. We hope that this protocol will serve as a resource to enhance the rigor and reproducibility of studies investigating innate and adaptive immune responses in the lungs.

## APPENDIX

### Fluorophore Panel Design Strategy for the Development of a Rigorous Protocol for Full Spectrum Flow Cytometry of the Lungs

#### Panel design steps.

1) The first step is to select the cell populations, lineage markers, and antibody clones for the panel (Supplemental Table S2). These will vary from study to study based on the scientific questions under investigation and the cell populations to be studied. For our studies, we undertook an unbiased approach with the goal to measure changes in the immune cells we knew could be present in the controls or recruited into the lungs of mice during the first 9 days of influenza infection (Fig. 1). When we identified the lineage markers of interest for our panel, we took note of the anticipated level of antigen expression for each marker and whether there was coexpression of that marker on multiple cell populations. These are important considerations during fluorophore selection (*steps 3* and *4*). Finally, antibody clones were selected based on their appropriateness for our mouse strain (*step 4*) and on published literature.
2) The second step of our panel design was to determine the autofluorescence (AF) signatures of each tissue. These were determined by evaluation of spectral signatures from unstained BAL, lung, and lymph node cells from control (PBS instilled) and influenza-infected mice collected at 3, 6, and 9 days after instillation (dpi) (Fig. 2 and Supplemental Figs. S6, S7, and S8). Heterogeneous AF spectra were analyzed and multiple populations of cells with high-intensity AF signatures in each tissue were identified as previously described (35). Briefly, we applied the N × N approach to raw spectral data from unstained samples. N is equal to 64, the number of fluorescence detectors on the Cytek Aurora 5 Laser 16UV-16V-14B-10YG-8R. Detector combinations (4,096) were observed for unstained cells from each tissue and treatment condition to find the combination that separated the greatest number of cell populations from one another with good resolution. Unique AF signatures were designated as fluorescent tags in the SpectroFlo library and given priority consideration during the assignation of fluorophores for other markers to the panel (*step 3*). It is critically important to avoid selecting fluorophores that share the same peak emission channels or that have similar spectral signatures to unique autofluorescence signatures. Therefore, assessing the complexity of heterogeneous AF is an essential spectral flow panel design step.
3) For the selection of specific fluorophore-antibody conjugates, fluorophores were assigned to channels with unique peaks and appropriate emission wavelengths (Supplemental Table S1). The following criteria were followed:
  3.1) Reagents that shared a peak channel with the Live/Dead Fixable Blue viability dye (UV6) were avoided.
  3.2) Reagents that shared peak channels with AF signatures were avoided.
  3.3) Reagents that had unique peak channels but shared highly similar spectra with AF signatures (e.g., AlexaFluor532 or BV570) were avoided as they yield poor resolution of cell populations due to spread into AF signatures.

Further considerations were given to balancing fluorophore stain index versus antigen expression level, minimizing the similarity and spread of fluorophores, and assignment of distinct fluorophores to coexpressed antigens. All fluorophore-antibody conjugates used for these studies were commercially available, and only surface antigens were chosen for detection.

4) Additional panel design considerations were made to accommodate dual in vivo CD45 labeling. The two antibodies chosen for dual in vivo labeling were of different clonality, recognizing different epitopes from the CD45 antibody chosen for in vitro labeling. In addition, the CD45.2 epitope was chosen for one of the in vivo epitopes due to its compatibility with the C57Bl/6J mice used in our study. This reduced the likelihood of saturating CD45 sites with the intravascular and intratracheal antibodies, which could diminish the intensity of in vitro staining. We assigned fluorophores with low-to-medium staining intensity to CD45 to reduce the likelihood of spillover and spread. This was important because CD45 is expressed at high levels on all leukocytes and coexpressed with all other markers in the panel. The fluorophores also had low similarity in their spectral profiles to ensure good resolution of leukocytes from different compartments. Preliminary experiments were conducted with the selected antibodies, AlexaFluor488-CD45.2 (IVCD45) and BUV615-CD45 (ITCD45), to establish titers yielding good resolution of IVCD45+ ITCD45− versus IVCD45− ITCD45+ versus IVCD45− ITCD45− populations in our gating strategy.
5) To assess potential spillover spreading in our panel that could contribute to unmixing errors, we evaluated our AF spectral signatures with all other selected fluorophores computationally using the Cytek Full Spectrum Viewer Similarity and Complexity matrix (Supplemental Fig. S1). Well-designed panels have similarity indices <0.98. Panels with a similarity index > 0.98 between two signatures will result in spillover spreading and unmixing errors. Well-designed small panels (e.g., 10 dyes or fewer) will have complexity indices around 2–3. Well-designed large panels (e.g., 35–40 colors) will have complexity indices around 40–50. Our panel’s complexity fell well below this benchmark due to our panel design strategy, which ensured we had fluorophore and autofluorescence spectral signatures that were very different from each other (similarity indices < 0.8). Evaluation of similarity and complexity indices is an important consideration when optimizing a spectral flow panel.
6) To optimize our panel to minimize nonspecific antibody-fluorophore binding and to obtain accurate resolution of fluorophores and autofluorescence signatures, all antibody-fluorophore conjugates were titrated using cells from 9 dpi IAV PR8-infected lung homogenate tissue with ∼100,000 cells per test (Supplemental Fig. S2). Conjugates were tested in twofold serial dilutions within the range of 2–2,000 ng per test, as indicated; preliminary tests were performed to determine the range to be evaluated for each conjugate. FlowJo was used to determine the separation index (SI), a metric for defining the separation between positive versus negative populations, for each dilution and to concatenate populations for visualization (51). Selected titers are reported in Supplemental Table S2. In some cases, the selected titer was not necessarily the one with the highest SI value; consideration was also given to the appropriate alignment of negative populations with zero median fluorescence intensity (MFI), adequate separation of positive and negative populations, and maintenance of positive populations below 10^6^ MFI.
7) To determine whether beads or cells would be the appropriate reference control material for each fluorophore-antibody conjugate in our panel, we determined the normalized full spectrum spectra for all of our reagents on both single stain beads and single stain cells from IAV PR8-infected lung homogenates as previously described (21). Discrepancies in spectra were found with the following fluorophores: BV421, BV711, BV750, and PerCP-eFluor 710. Therefore, these four fluorophores were run as single-stain controls using cells for our experiments while all remaining fluorophore single-stain controls were run using beads. The live/dead viability reference control was based on a mixture of live and heat-killed cells. The resolution of every fluorophore in the panel was checked by comparing the single stain control tube with the same fluorophore in the full-stained sample. We found that the resolutions were identical and no changes to the selected titrations were made.

#### Preparation of reagents used for lung digestion.

A stock solution of Liberase (Sigma-Aldrich, St. Louis, MO) was prepared at 10 mg/mL (26 U/mL) in RPMI 1640 without phenol red or fetal bovine serum (FBS) (Thermo Fisher Scientific Waltham, MA), stored in single-use aliquots at −30°C, and used by the expiration date per the manufacturer’s recommendation. A stock solution of recombinant DNase I (Sigma-Aldrich, St. Louis, MO) was prepared at 10,000 U/mL in H_2_O and stored in single-use aliquots at −30°C for up to 1 mo. Just before harvest, a working solution of enzyme digestion mixture, containing Liberase TM (0.26 U/mL) and recombinant DNase I (10 U/mL), was prepared in RPMI 1640 without phenol red or FBS in a volume of 2 mL per mouse and stored on ice until needed for enzymatic digestion of lungs.

#### Protocols for In vitro antibody staining.

The following reagents were prepared for in vitro antibody staining:

1) Fc Block TruStain FcX PLUS (anti-mouse CD16/32) (BioLegend, San Diego, CA) was diluted 1:100 (vol:vol) with Flow Cytometry Staining (FCS) buffer (Thermo Fisher Scientific Waltham, MA) in a total volume of 50 µL per test and stored on ice until use.
2) Just before use, one vial of LIVE/DEAD Fixable Blue Dead Cell Stain Kit (Thermo Fisher Scientific Waltham, MA) was resuspended with 50 µL DMSO and then diluted 1:320 (vol:vol) in PBS in a total volume of 20 µL per test. LIVE/DEAD Blue Cell Stain must be prepared in a protein-free solution, without FCS, as protein can impact the efficiency of LIVE/DEAD Blue staining
3) To prepare the full-stain antibody master mix, all antibodies were first gently vortexed and centrifuged at 10,000 *g* for 3 min at 4°C. Antibodies were diluted in Brilliant Stain Buffer (BD Biosciences San Jose, CA) to achieve the final titers indicated in Supplemental Table S2, allowing for a total volume of 100 µL per test. The antibody master mix excluded the antibody conjugates used for in vivo staining, Alexa Fluor 488 anti-CD45.2 (IVCD45) and BUV615 anti-CD45 (ITCD45), and was stored on ice until use.

The following cells and beads were prepared for in vitro staining:

1) For full-stained samples, up to 1 × 10^6^ cells from BAL fluid, lungs, and lymph nodes of mice that received both IVCD45 and ITCD45 were used.
2) Tissue from a mouse that did not receive IVCD45 or ITCD45 was used for preparation of unstained controls, the single-stain viability control, and single-stain cell controls (CD11c-BV421, CD64-BV711, SiglecF-BV750, and γδTCR-PerCP-eFluor 710).
  2.1) For unstained controls, up to 1 × 10^6^ cells from BAL fluid, lungs, and lymph nodes were used.
  2.2) For the viability control, 2.5 × 10^5^ lung cells were heat-killed for 10 min at 75°C, and after chilling for 10 min on ice were combined with 2.5 × 10^5^ additional lung cells.
  2.3) For each single-stain cell control, 2.5 × 10^5^ cells from the lungs were used.
  2.4) Single-stained controls for the remaining fluorophore-antibody conjugates were prepared using UltraComp eBeads (Thermo Fisher Scientific Waltham, MA), following the manufacturer’s instructions.

The following steps were followed for in vitro staining:

1) In vitro antibody staining was performed in a 96-well V-bottom plate (Thermo Fisher Scientific Waltham, MA).
2) The appropriate number of cells and beads were aliquoted and pelleted via centrifugation at 400 *g* for 3 min at 4°C.
3) The supernatant was carefully removed, and all samples were washed with 200 µL of PBS.
4) Samples were pelleted, supernatant removed, and samples were resuspended with 100 µL of PBS.
5) Viability dye dilution (20 μL) was added to viability control and full-stained samples, mixed well, and incubated for 15 min at room temperature in the dark.
  5.1) Unstained and single-stained controls received 20 µL of PBS instead of viability dye dilution.
6) After incubation, 100 µL of FCS buffer was added to all samples.
7) Samples were pelleted, supernatant removed, and samples were resuspended with 200 µL of FCS buffer.
8) Samples were pelleted, supernatant removed, and 10 µL of brilliant stain buffer was added to all full-stained samples and mixed well.
9) Fc Block (50 µL) was added to all samples and incubated for 5 min at room temperature in the dark.
10) Full-stain antibody master mix (100 µL) was added to samples for full staining.
  10.1) FCS buffer (100 µL) was added to unstained controls and viability control.
  10.2) Single-stained cell controls (CD11c-BV421, CD64-BV711, SiglecF-BV750, and γδTCR-PerCP-eFluor 710) received the appropriate titer of their designated fluorophore-antibody conjugate as indicated in Supplemental Table S2.
  10.3) Single-stain bead controls received 0.5 µL of their fluorophore-antibody conjugate diluted in 100 µL of FCS.
11) All samples were incubated for 30 min at room temperature in the dark, after which 100 µL of FCS buffer was added to all samples.
12) Samples were centrifuged, supernatant removed, and samples were resuspended with 200 µL of FCS buffer.
13) Samples were pelleted, supernatant removed, and samples were resuspended with 100 µL of Cytofix (BD Biosciences, San Jose, CA).
14) All samples were incubated for 15 min at room temperature in the dark, after which 100 µL of FCS buffer was added to all samples.
15) The samples were centrifuged, supernatant removed, and samples were resuspended with 200 µL of FCS buffer.
16) The samples were centrifuged again, supernatant removed, and samples were resuspended with 200 µL of FCS buffer.
17) Finally, samples were transferred to 5-mL tubes (VWR, Brisbane, CA) and stored overnight at 4°C in the dark until acquisition on the flow cytometer.

#### Data acquisition for flow cytometry.

The optical design, detection modules, number of detectors per laser module, detector module configurations, and methodology for establishing the optimal instrument gain settings, known as CytekAssaySetting, for commercially available fluorophores have been previously reported (21). For our studies, we used CytekAssaySetting, which automatically updates gain settings during daily quality control (QC) on calibrated bead MFI targets to ensure consistent instrument setup over time. Before all data collection, QC on calibrated beads was performed and data were only collected when the instrument passed QC.

It was imperative to ensure our samples were on scale as our panel was designed to detect relatively small cells such as lymphocytes and very large cells such as alveolar macrophages. To address this, the FSC, SSC, and SSC-B gain settings were adjusted using tissue-specific unstained samples immediately before acquisition. SSC-B-A was set to a log scale to improve visualization of especially large cells, such as alveolar macrophages, on SSC-B-A plots. FSC area scaling factor was reduced to 0.9 in CytekAssaySetting. For each tissue type, FSC, SSC, and SSC-B gain settings were adjusted immediately before data acquisition to ensure >95% of all cells were on scale. The FSC threshold was set at 250,000. Acquisition stopping gates were set to 200,000 events or 125 µL and were recorded at low-medium flow.

#### Spectral unmixing.

Raw data were converted to unmixed data with SpectroFlow software v3.0 using an ordinary least-squares algorithm to deconvolute individual fluorophore signatures within a fully stained sample. As part of our assessment of our panel’s performance, we compared spectral unmixing of our panel with and without AF spectral signatures that initially presented as heterogeneous AF in our unstained samples. Identification of AF signatures was necessary for lung cells and was dependent on the day after instillation. AF signatures were saved as discrete fluorochrome tags in the SpectroFlo library as previously described. These were then incorporated into our panel as single-stain reference controls for unmixing and data analysis. It was not necessary to define AF signatures for lymph nodes as cells from these compartments had low, homogeneous AF over the course of these studies.

In both unmixing strategies, we used the “AF as a tag” (AF) function in the SpectroFlo (Cytek Biosciences, CA) software. As with fluorescent dyes, AF is susceptible to spillover interactions with other fluorophores that have emission spectra in similar wavelength ranges. This was evaluated computationally using the Cytek Full Spectrum Viewer Similarity and Complexity Index functionality. The 21 fluorophore panel presented here has a low predicted complexity index of 7.46 (not shown). Inclusion of two unique AF signatures for lungs of 9 dpi PR8 mice increases the complexity index only to 10.21 (Supplemental Fig. S1). In addition, we visualized unmixing errors in both unmixing strategies by evaluating N × 1 permutations of all fluorophores in the panel against AF on unstained lungs from a mouse 9 dpi with IAV PR8. Finally, we applied our supervised gating strategy (Fig. 1, Table 1, and Supplemental Figs. S3, *A–D*, S4, *A–D*, and S5, *A* and *B*) to both unmixing strategies and compared cell counts for individual cell populations from both unmixing strategies.

## DATA AVAILABILITY

Data will be made available upon reasonable request.

## SUPPLEMENTAL DATA

10.6069/ARQ3-E011 Supplemental Tables S1 and S2 and Supplemental Figs. S1–S9: https://doi.org/10.6069/ARQ3-E011.

## GRANTS

This work was supported by NIH Grants R01AI130280, R01AI136468, and R21AI147536.

## DISCLOSURES

No conflicts of interest, financial or otherwise, are declared by the authors.

## AUTHOR CONTRIBUTIONS

M.Y.C., J.E.B., and C.W.F. conceived and designed research; M.Y.C. and J.E.B. performed experiments; M.Y.C., J.E.B., and M.B. analyzed data; M.Y.C., J.E.B., M.B., W.A.A., and C.W.F. interpreted results of experiments; M.Y.C. and J.E.B. prepared figures; M.Y.C. and J.E.B. drafted manuscript; M.Y.C., J.E.B., W.A.A., and C.W.F. edited and revised manuscript; M.Y.C., J.E.B., M.B., W.A.A., and C.W.F. approved final version of manuscript.
